# The anatomo-functional connectivity of word repetition: insights provided by awake brain tumor surgery

**DOI:** 10.3389/fnhum.2013.00405

**Published:** 2013-07-29

**Authors:** Sylvie Moritz-Gasser, Hugues Duffau

**Affiliations:** ^1^Department of Neurosurgery, Hôpital Gui de Chauliac, Centre Hospitalier Universitaire MontpellierMontpellier, France; ^2^Team “Plasticity of Central Nervous System, Stem Cells and Glial Tumors,” INSERM U1051, Institute for Neuroscience of Montpellier, Hôpital Saint EloiMontpellier, France

Word repetition (WR) is the language process which consists of immediately saying a word after hearing it. This skill plays a crucial role in language development by enabling the learning of new words. Clinically, repetition skill participates in the classification of aphasic syndromes, and can inform about prognosis (Hosomi et al., 2009) and guide rehabilitation (Schlaug et al., 2009). In addition, uncontrolled pathological repetition termed echolalia is observed in neurological disorders such as autism.

Despite improving knowledge of cortical networks underlying WR, its subcortical connectivity has received less attention. Here, we investigate the white matter pathways involved in WR.

## Neuropsychological considerations

WR involves several levels of linguistic processing. The conversion of phonological information into articulatory-based representations requires multiple steps: (1) phonological decoding and temporary storage within the verbal working memory system utilizing subvocal rehearsal (Baddeley, 1996); (2) semantic recognition as word versus pseudo word; (3) phonological encoding and (4) planning for execution by the motor articulatory programs. These parallel and distributed levels of processing are supervised by initiation and inhibitory cognitive mechanisms.

The debate about the respective contributions of phonological and lexical-semantic representations in auditory WR remains open. WR impairment may be explained by a “dual-route” model in which non-lexical and lexical-semantic pathways are distinct with some degree of interaction (Hanley et al., 2002). An alternative explanation is the “single-route” model based on a “globality assumption,” in which phonological and lexical-semantic representations interact in all aspects of repetition (Dell et al., 1997). In clinical practice, phonological errors during immediate repetition are regularly observed in patients harboring lesions involving the semantic system while to a lesser extent semantic errors may be observed during immediate repetition in patients with damage to the phonological system. Even though these observations support that semantic and phonological representations interact within a large-scale network, WR neural foundations remain poorly understood.

## Neural bases of word repetition: the classical dorsal stream

Wernicke (1874) described a region within the left posterior temporal lobe devoted to auditory word processing and postulated the existence of a connection between this area and the inferior frontal gyrus (IFG). He suggested that a lesion of this connection induced, in addition to phonemic paraphasias with associated correction strategies, the inability to repeat. The white matter pathway underlying this connection has been referred as the arcuate fasciculus (AF), and language disorders elicited by damage to the AF were termed “conduction aphasia” (Goodglass, 1992). Geschwind (1965) argued that this disconnection syndrome may be caused by a lesion to either the AF or to the left inferior parietal lobe (IPL). Advances in neuroimaging combined with lesion studies have confirmed the involvement of the supramarginalis gyrus (SMG) within the IPL in WR (Fridriksson et al., 2010), in addition to the IFG and cortical areas surrounding the posterior portion of the Sylvian fissure (Damasio and Damasio, 1980; Price et al., 1996; Baldo and Dronkers, 2006; Buchsbaum et al., 2011). In particular, a small temporal-parietal area (“area Spt” for Sylvian-parietal-temporal), inferior to the SMG, is thought to be crucial in phonological working memory and WR (Baldo et al., 2011). According to the dual stream model of language processing, the area Spt might represent an “interface” for the integration of sensorimotor representations of verbal sounds. Thus, lesions to Spt may result in inefficient transfer of auditory representations into articulatory representations stored in the IFG and ventral premotor cortex. The described temporal-parietal-frontal network would constitute a dorsal language stream dedicated to phonological processing, i.e., the ability to link sound to articulation—while a ventral stream would be dedicated to semantic processing (linking sound to meaning) (Hickok and Poeppel, 2004). It is worth noting that Wernicke himself assumed the existence of a ventral stream in addition to the dorsal one, running behind the insula and connecting temporal and frontal regions. However, this very modern view fell into oblivion up to the recent highlighting of such a dual stream organization of language (Weiller et al., 2011).

Recent studies combining functional MRI and diffusion tensor imaging show that this dorsal stream is subserved by the superior longitudinal fasciculus (SLF) (Catani et al., 2005; Saur et al., 2008). The SLF is comprised of three components: a medial direct pathway corresponding to the AF and two indirect pathways running laterally: an anterior part connecting the ventral premotor cortex with the inferior parietal cortex and a posterior part connecting the inferior parietal cortex with posterior temporal areas (Catani et al., 2005). In this dual stream model, the major role of a left dorsal network in WR, connecting via the AF/SLF complex, posterior temporal, inferior parietal, and inferior frontal areas, is now admitted (Fridriksson et al., 2010).

The question remains as to whether the dorsal stream is sufficient for WR or whether the ventral pathway can impact WR, as was recently suggested (Berthier et al., 2012). Intraoperative language mapping performed during awake brain tumor surgery, combined with perioperative language evaluations, may provide significant insights into this question.

## Intraoperative mapping and perioperative examinations: does the ventral stream play a role in word repetition?

### Intraoperative language mapping

Awake surgery for tumor patients allows maximizing the extent of resection while preserving brain functions (Duffau, 2005). Direct electrical stimulation (DES) is applied over the brain, which temporarily inactivates restricted structures both at cortical and subcortical levels. Sensorimotor and language functions are continuously assessed throughout the resection. If a disorder is reproducibly observed during DES of a given area, that area is regarded as critical to the functional network being evaluated and is thus preserved. Based on an experience with hundreds of procedures, awake surgery enables mapping of eloquent pathways in humans with a spatiotemporal resolution unmatched by other techniques (Duffau et al., 2008). In practice, language is evaluated using a picture naming test: the patient is asked to name black and white pictures of common objects that are presented on a computer screen. When transient language disorders (such as phonological paraphasias, semantic paraphasias or anomias) are generated by DES, WR is assessed by simply asking the patient to repeat the correct word.

Here we report a summary of the more frequent disorders observed during intraoperative DES in 200 patients undergoing awake surgical procedures for brain tumors between 2009 and 2012. Reproducible disturbances were observed in approximately 80% of cases, demonstrating a reliable methodology despite interindividual variability and brain reorganization induced by the glioma (Table 1).

**Table 1:** **Neural bases of word repetition**.

			**Cortical level**			**Subcortical level**		
			**vPMC**	**inf SMG**	**pSTG/pMTG**	**sup pAF**	**lat vSLF**	**IFOF**
	DES	Naming	sp. arrest	Anomia + Pho P	Anomia	Pho P	Artic disord.	Sem P
		Word repet	sp. arrest	Pho P	OK	Pho P	Artic disord.	OK or *Perse*
**Naming**	**Phonological network**				**Semantic network**			
	**OK**	**Anomia**	**Pho P**	**Artic disord.**	**OK**	**Anomia**	**Sem P**	**Sem P**
	↓	↓	↓	↓	↓	↓	↓	↓
Real word repet	OK	OK/Pho P	OK/Pho P	Artic disord.	OK	OK/Pho P/Sem P	OK/*perse*/Pho P	OK/Pho P
Pseudo word repet	OK/Pho P	Pho P/Ø	Pho P/Ø	Artic disord./Ø	OK/Pho P/regul	OK/Pho P/regul	OK/regul/Pho P/Ø	Pho P/Ø

Upper: Language disorders induced by intraoperative DES of brain areas of interest during picture naming, and then followed by word repetition. Lower: Performances on the immediate postoperative language evaluation, likely reflecting edema-induced disruption of functional networks (phonological/semantic) adjacent to the surgical cavity. Abbreviations: DES, direct electric stimulation; word repet, word repetition; sp. arrest, speech arrest; Pho P, phonological paraphasia; artic disord., articulatory disorder; Sem P, semantic paraphasia; Perse, perseveration; vPMC, ventral premotor cortex; inf SMG, inferior supramarginalis gyrus; pSTG, posterior superior temporal gyrus; pMTG, posterior middle temporal gyrus; sup pAF, postero-superior arcuate fasciculus; lat vSLF, lateral ventral part of the superior longitudinal fasciculus; IFOF, inferior fronto occipital fasciculus; Ø, absence of response; regul, regularization (transformation of a pseudo word into a real word).

At the cortical level, DES of the inferior part of the SMG induced anomias or phonological disorders during the picture naming test. During WR tasks, phonological paraphasias were observed. DES of the ventral premotor cortex induced always a speech arrest, during both naming and WR. DES of the posterior part of the superior and middle temporal gyri induced anomias, with preservation of WR.

At the subcortical level, DES of the AF reliably induced phonological disorders during both naming and WR. Moreover, DES of the anterior part of the lateral SLF induced articulatory disorders during naming and WR. Finally, DES of the inferior fronto-occipital fasciculus (IFOF) produced semantic paraphasias related to the target word (i.e., belonging to the same category than the target word, or having an associative link with it) during naming, whereas during WR, the patient repeated normally or perseverated on the previous semantic paraphasia.

Our observations support the dual stream model of language processing for both naming and WR. First, the dorsal phonological stream is supported by two components of the SLF: the longer, more medial AF which is crucial for decoding and encoding phonological representations (i.e., the phonological buffer), and the antero-lateral SLF III (Makris et al., 2005), which is essential in linking phonological information to articulatory-based representations i.e. the articulatory loop of verbal working memory (Duffau et al., 2003; Maldonado et al., 2011). In a recent study that utilized fiber dissection and tractography to isolate the subcomponents of the SLF and to identify their cortical terminations, it was demonstrated that (1) the AF connects the middle and inferior temporal gyri with the precentral and inferior frontal gyri, (2) the anterior segment of the SLF (SLF III) connects the SMG and superior temporal gyrus with the precentral gyrus (Martino et al., 2013). AF/SLF III connects different frontal, temporal and parietal areas that were previously shown to be critically involved in WR. Thus, the critical role of the dorsal stream in WR was validated by intraoperative DES. Concerning the ventral semantic stream, we have previously demonstrated the underlying role of the IFOF, where stimulation of this fascicle systematically induced semantic disorders during naming (Duffau et al., 2005; Duffau, 2008). Interestingly, WR can be unaffected or impaired (perseveration) during IFOF stimulation.

In summary, these intraoperative stimulation data indicate that when the AF/SLFIII is transiently inhibited by DES, inducing a disruption in the connectivity of the dorsal phonological network, WR is inefficient. When the ventral semantic network is inhibited via IFOF stimulation, WR can be either completely unaffected or disrupted, resulting in perseveration. Therefore, it seems that although the dorsal phonological network is crucial for WR, the ventral semantic network may play a role in control mechanisms of semantic activations during WR.

### Perioperative language assessments

Per our standard protocol, every patient undergoing awake surgery for glioma resection has preoperative (day prior to surgery) and postoperative (5 days and 3 months following operation) language evaluations. These evaluations include:

- The same naming task as intraoperatively (Metz-Lutz et al., 1991),
- A verbal fluency task (phonological and semantic) (Cardebat et al., 1990),
- A non-verbal semantic association task (Howard and Patterson, 1992),
- A reading task (words and pseudo-words) (personal material).

Preoperative examination allows assess- ment of the impact of the tumor on language functions. Immediate postoperative evaluation is particularly informative, because the postsurgical edema surrounding the surgical cavity may induce transient functional disorders (a few day) thereby illustrating the functional role of brain regions near the surgical cavity. Finally, the language examination 3 months after resection enables assessing the efficiency of functional reorganization following speech rehabilitation.

Preoperative evaluations showed no significant deficit.

### Postoperative evaluations

During the immediate postoperative evaluation of picture naming, as during surgery, when a disorder was observed, the patient was asked to repeat the correct word. Furthermore, at the end of the assessment, all patients were asked to repeat, with only auditory input, a series of simple real words and pseudo-words (Table 1).

Patients in whom the phonological network was primarily involved by postoperative edema (parietal operculum, parietal-posterior temporal lesions) presented more often with WR disorders than patients in whom the semantic network is mostly involved (frontal-temporal, inferior temporal lesions). Typically, real word and pseudo-WR gave rise to phonological or articulatory disorders, suggesting that when the connectivity underlying the phonological system is transiently damaged, the semantic network alone is generally not able to compensate for the phonological stream. Nonetheless, in some cases, repetition of real words was possible even in the presence of naming disorders. Therefore, we suggest some degree of participation of the semantic system in real WR.

Patients in whom the semantic network was primarily involved by postoperative edema (frontal-temporal, inferior temporal lesions) rarely experienced repetition disorders. In these rare cases, the most frequent disorder was phonological paraphasia, indicating that when the connectivity underlying the semantic system is transiently damaged, the phonological network may sometimes not be able to link sound to articulation for accurate WR. Another disorder observed was the regularization of pseudo-words, i.e. the production of a real word with phonological similarities, suggesting that the inefficiency of lexical judgment due to semantic disorganization is not compensated by the phonological processing.

Three months after surgery, following intensive speech-therapy, most patients recovered normal WR performance. The few patients who presented with persistent WR disorders were those with cavity or residual tumor involving the phonological network—but not the semantic network.

In summary, despite the crucial role of the phonological stream in repetition of real words or pseudo words, these observations underline strong interactions between the phonological and semantic systems. This is in agreement with previous studies highlighting on the one hand a *“division of labour”* between the two pathways (this division being not mutually exclusive, especially in the context of post-stroke recovery), and on the other hand the *“interactive contributions of the two pathways”* in single-word comprehension and production, including WR (Nozari et al., 2010; Ueno et al., 2011).

## Conclusion

The dorsal phonological pathway, supported by the left AF/SLF complex subcortically, is crucial to accurate WR. It enables the conversion of auditory input, processed in verbal working memory system, into phonological and articulatory-based representations. Although this contribution is essential, the ventral semantic pathway, connected by the left IFOF, also contributes to WR of real words or pseudo-words. Indeed, perioperative and intraoperative language evaluations of gliomas patients undergoing awake surgery highlight the strong interaction of both pathways in WR in order to convert auditory input into articulatory output efficiently.

Further studies are needed to validate this dual stream model of WR, and to bring insights into the possible role of the right hemisphere.
